# Antibacterial and antibiofilm activity of *Eucalyptus globulus* leaf extract, asiatic acid and ursolic acid against bacteria isolated from bovine mastitis

**DOI:** 10.3389/fvets.2025.1565787

**Published:** 2025-05-14

**Authors:** Nicolò Mezzasalma, Costanza Spadini, Chiara Spaggiari, Giannamaria Annunziato, Valentina Andreoli, Alice Prosperi, Lorenzo Mochen, Sandro Cavirani, Stefano Grolli, Simone Taddei, Gabriele Costantino, Clotilde Silvia Cabassi

**Affiliations:** ^1^Department of Veterinary Science, University of Parma, Parma, Italy; ^2^Department of Food and Drug, University of Parma, Parma, Italy; ^3^Istituto Zooprofilattico Sperimentale della Lombardia e dell’Emilia-Romagna (IZSLER), Brescia, Italy

**Keywords:** plant extracts, pentacyclic triterpenes, biofilm-producing organisms, multidrug-resistant organisms, MIC, minimal biofilm inhibitory concentration

## Abstract

Antibiotics represent the first line therapy for bovine mastitis. However, the increasing prevalence of multidrug-resistant organisms (MDROs) highlights the need for alternative therapeutic approaches. This study evaluated the antimicrobial and antibiofilm activities of *Eucalyptus globulus* leaf extract (EGL-L), ursolic acid (UA) and asiatic acid (AA) against *Staphylococcus aureus* (SA), *Streptococcus uberis* (SU), *Streptococcus agalactiae* (SAG), and *Enterococcus* spp. (EN) isolated from bovine mastitis, 39.7% of which were MDROs. The minimal inhibitory concentration (MIC) assay demonstrated that all the compounds exhibited antimicrobial activity against the tested bacteria, including MDROs. However, EGL-L was less effective (*p* < 0.001) than UA or AA against field strains. UA was more effective against SAG and SU compared to SA (*p* < 0.001), whereas AA was more effective against SU than SA (*p* < 0.001). Conversely, EGL-L exhibited similar inhibitory effects on all bacteria. The biofilm-forming ability of the bacterial strains was also assessed, and the minimal biofilm inhibitory concentrations (MBICs) of the compounds were evaluated for moderate and strong biofilm producers. None of the compounds were able to completely inhibit biofilm formation. However, MBIC_80_ values within the tested concentration range were achieved for 15 out of 32 strains with EGL-L and for 27 out of 32 strains with UA and AA. These findings highlight a promising alternative to conventional antimicrobials for AA and UA, showing potential for topical intramammary use for the control and prevention of bovine mastitis, especially because of their efficacy against biofilm formation. Future research should focus on toxicity assessments and formulation development for potential topical administration.

## Introduction

Bovine mastitis (BM) is an inflammation of the mammary gland caused by physical trauma or microorganisms that can affect the health and welfare of animals (1). In dairy cow husbandry, it is the most important cause of increased farm costs because of reduced milk production, increased medical expenses and increased animal culling (2). Contagious mastitis is caused by bacteria that recognize the udder as a primary reservoir (e.g., *Streptococcus agalactiae* and *Staphylococcus aureus*). On the other hand, environmental mastitis is commonly caused by bacteria found in the environment or fecal matter, especially under poor hygiene conditions (e.g., *Escherichia coli* or *Streptococcus uberis*) (3).

Antibiotics represent the first-line therapy for bovine mastitis, and intramammary administration (IMM) is preferred (4). However, the effectiveness of IMM therapy could be hindered by the intracellular localization of bacteria or the low lipid solubility of antimicrobial molecules, which predisposes bacteria to the emergence of antibiotic resistance, and by the ability of some bacteria to form biofilms (5). One of the key strategies for mastitis control is dry-cow therapy (DCT), which involves the intramammary administration of long-acting antimicrobial agents during the dry-off phase (6). DCT can be performed on all quarters of all drying cows (blanket DCT) or only on infected cows or quarters (selective DCT) (7). The blanket DCT was the most widely used strategy in the past to manage BMs (8). However, the worldwide increase in AMR among mastitis-associated bacteria poses significant threats to both animal and human health (9). The selective DCTs reduce antibiotic use, counteracting the spread of antimicrobial resistance. Nevertheless, a high incidence of new intramammary infections has been reported in untreated dry cows without clinical signs of mastitis (7, 10). In accordance with [Regulation (EU) 2019/6 of the European Parliament and the Council], the use of antibiotics in veterinary medicine is rigorously regulated (11). Therefore, the development of innovative approaches is imperative to achieve effective disease control while simultaneously reducing antibiotic usage.

The most investigated alternative strategies are based on probiotics, bacteriophages and animal-, plant-, and bacteria-derived antimicrobials (4). Plant derivatives, particularly plant extracts and essential oils, have been widely used in traditional medicine and have recently gained attention in veterinary medicine because of their biological properties, including antimicrobial activity (12–14). Compared with conventional antibiotics, essential oils and plant extracts offer several advantages: (i) they are classified as nonpharmaceutical compounds, (ii) they have few side effects, and (iii) their prolonged use is not associated with the development of resistance (1, 6). As these molecules act on different targets than antibiotics do, often different components of the bacterial wall, they can also be used to treat infections caused by multidrug-resistant organisms (MDROs) (6, 9). An interesting and promising product of plant origin is the extract of *Eucalyptus globulus* Labill. whose chemical composition is widely documented in the literature, along with its antibacterial and antifungal properties (15, 16). Its chemical composition, and consequently its biological properties, may vary depending on the geographical origin of collection. This variation can be influenced by environmental factors (e.g., soil composition and climate), leaf age, and genetic variations (17). Indeed, the antimicrobial effects of *Eucalyptus globulus* are primarily attributed to its phenolic compounds and pentacyclic triterpenes, which demonstrate greater efficacy against Gram-positive bacteria compared to Gram-negative bacteria. This difference in effectiveness is likely due to the protective outer membrane lipopolysaccharide layer of Gram-negative bacteria. The main active compounds specifically target cellular structures in a nonspecific manner, with proposed mechanisms including alterations in membrane permeability, loss of membrane potential, dysfunction of the proton pump, and depletion of ATP (18).

Among the most bioactive compounds of interest is the triterpene family, particularly pentacyclic triterpenes such as asiatic acid (AA), ursolic acid (UA), and their derivatives, which are known for their antibacterial properties against Gram-positive and Gram-negative bacteria (16, 19, 20). Pentacyclic triterpenes represent the most abundant group of terpenoids found in dicotyledons and serve as chemical defenses against competing plants, pathogens and herbivores. They exhibit antioxidant, antimicrobial, fungicidal and antiparasitic properties (19). As extensively reviewed in literature (19), the antibacterial activity of pentacyclic triterpenes is associated with alterations in bacterial cell structure, stimulating chemotaxis-related genes involved in host defense and affecting bacterial gene expression related to biofilm formation, peptidoglycan turnover, and cell autolysis. Additionally, the review mentioned highlights that both acids show higher antibacterial activity against Gram-positive bacteria rather than Gram-negative, probably due to the difficulty of these acids to overcome the outer membrane of Gram-negative. Consequently, there are differences in AA and UA concentrations required to inhibit bacterial growth in Gram-positive and negative bacteria. Ursolic acid is a constituent of several medicinal plants and it is known for its wide range of biological properties such as antioxidant, antibiofilm and antibacterial activities, especially against Gram-positive bacteria (20, 21). In the literature, the ability of UA to inhibit *E. coli* biofilm formation under different conditions has been described (24) influencing its transcriptome, including gene repression of *CysB* and *CysDJK* of the bacterial biosynthetic way of cysteine, which are involved in the response to oxidative stress and biofilm formation (15, 25, 26). Asiatic acid is instead known for its antimicrobial activity against both Gram-positive and Gram-negative bacteria (16, 19). However, the number of studies reporting its antibiofilm activity is lower than that reporting its activity toward UA (26).

However, few studies have focused on the antimicrobial and antibiofilm activities of AA, UA and *Eucalyptus globulus* Labill. leaves extract (EGL-L) against bacteria involved in BM.

Therefore, the present study aimed to quantify the content of AA and UA in EGL-L strains originating from Rwanda and subsequently evaluate their antimicrobial activity against both reference and field strains of *S. aureus*, *S. agalactiae*, *S. uberis* and *Enterococcus* spp. isolated during clinical and subclinical bovine mastitis. In addition, the antibiofilm activity of these alternative compounds was assessed specifically on moderate and strong biofilm-producing strains.

## Materials and methods

### Chemicals and reagents

Liquid chromatography–mass spectrometry (LC–MS)-grade methanol and acetonitrile were purchased from Scharlab Italia srl (Milan, Italy); distilled water was obtained via a Milli-Q system (Millipore, Bedford, MA, USA). MS-grade ammonium acetate, acetic acid, and formic acid from Fisher Chemical (Thermo Fisher Scientific Inc., San Jose, CA, USA) were also used. Ursolic and asiatic acid <98% (HPLC) were purchased from Sigma–Aldrich (Schnelldorf, Germany).

### Plant material

Leaves of *Eucalyptus globulus* (Labill., 1800) (EGL-L) were collected from eight different plants grown in the botanical garden of the INES Ruhengeri Institute of Applied Sciences, Musanze, Rwanda. Leaves were washed with tap water and air dried for 3 weeks. After drying, the leaves were ground using a grinder (particle size <800 μm).

### EGL-L extract preparation

Three aliquots of *Eucalyptus* leaves (20 g each) were shredded, placed into separate hermetic flasks and subjected to hydroalcoholic maceration with 70% ethanol. A 1:10 matrix-to-solvent ratio was used. The extraction process was carried out for 72 h at room temperature (RT) with constant stirring at 100 rpm. After extraction, the extracts were filtered, and the solvent was removed via a rotary evaporator (Buchi). The resulting extracts were frozen with liquid nitrogen and then freeze-dried. The lyophilization process was conducted under vacuum conditions for 36 h at −56°C and 1 mbar (1-DL alpha Plus freeze-drier). The dried extracts were divided into different aliquots and reconstituted in EtOH:water (70,30) prior to LC–MS analysis.

### HPLC–MS method for the quantification of UA and AA in EGL-L

The quantification of UA and AA in EGL-L was conducted via HPLC–MS in SIM mode. The analyses were performed in triplicate to ensure accuracy and reproducibility. Calibration curves were constructed for both compounds, which were subsequently used to determine their concentrations in the extract samples. HPLC–MS analysis was performed using a 2,695 Alliance separation system (Waters Go, Milford, MA, USA) equipped with a QuattroTM API triple quadrupole mass spectrometer with an electrospray source (Micromass, Waters, Manchester, UK). Chromatographic conditions were the following: Column XSelect®HSST3 (250 mm × 2.1 mm, 5 μm), flow rate 0.2 mL/min, column temperature 30°C, injection volume 5 μL. A gradient profile was applied using water (eluent A) and acetonitrile (eluent B) as mobile phases both acidified with 0.1% formic acid. The initial conditions were set at 100% A, after 5 min of the isocratic step, a linear change to 100% B at 8 min, and holding for 7 min before returning to initial conditions. Columns recondition was achieved over 6 min, providing a total run time of 21 min. The column was maintained at 30°C and a flow rate of 0.20 mL/min was used. MSD parameter: ESI negative, capillary voltage 2.5 kV, cone voltage 25 V, extractor voltage 2, source block temperature 120°C, desolvation temperature 350°C. Cone-gas-flow nitrogen and argon were used as collision gas. Selective ion monitoring (SIM) in negative ion mode was used. Ursolic acid was recorded as m/z 455.4 [M-H]^−^ while asiatic acid as 487.7 [M-H]^−^. UA and AA could not be collided into fragments when collision energy was 40 eV, or no dominant product ions were detected if collision energy was higher than 50 eV, which indicated that MRM experiment was not suitable for UA and AA quantification. Analytes concentrations in the sample were calculated from the relation within slope line obtained by linear regression analysis of this calibration curve and multiplied for their dilution factor.

### Bacterial strains

The following reference strains were tested: *Staphylococcus aureus* ATCC 25923, methicillin-resistant *Staphylococcus aureus* ATCC 43300 (MRSA), *Streptococcus agalactiae* ATCC 27956, *Streptococcus uberis* ATCC 19496 and *Enterococcus faecium* ATCC 19434.

Clinical isolates of *Staphylococcus aureus* (SA; *n* = 15), *Streptococcus agalactiae* (SAG; *n* = 17), *Streptococcus uberis* (SU; *n* = 18), and *Enterococcus* spp. (EN; *n* = 13), obtained from cows with subclinical or clinical mastitis, were tested.

Clinical strains were provided by the biobanks of the Animal Infectious Disease Laboratory of the University of Parma and by the IZSLER (*Istituto Zooprofilattico Sperimentale della Lombardia e dell’Emilia-Romagna*) – Laboratory of Parma.

Bacterial strains were grown in Columbia blood agar after incubation of 24 h at 37°C. On individual colonies, Gram staining and catalase test were performed, followed by execution of API Staph® and API 20 Strep® System test (bioMérieux), as well as indicated by manufacturer (27).

### Antimicrobial susceptibility testing (AST)

AST was performed via the Kirby–Bauer disk diffusion method. Owing to the intrinsic resistance of *Enterococcus* spp., testing for this genus was limited to the following antibiotics: amoxicillin/clavulanic acid (20/10 μg), ampicillin (10 μg), enrofloxacin (5 μg), erythromycin (15 μg), florfenicol (30 μg), imipenem (10 μg), marbofloxacin (5 μg), oxytetracycline (30 μg), penicillin G (10 U), rifaximin (40 μg), and vancomycin (30 μg). For the strains belonging to the *Streptococcus* genus, in addition to the abovementioned antibiotics, the following antibiotics were also tested: cefazolin (30 μg), cefquinome (30 μg), ceftiofur (30 μg), cefuroxime (30 μg), and trimethoprim/sulphamethoxazole (1.25/23.75 μg). *Staphylococcus aureus* isolates were tested with all the antibiotics listed above, except vancomycin. Additionally, they were tested with fusidic acid (10 μg), gentamicin (10 μg), kanamycin (30 μg) and lincomycin (15 μg). The interpretation of the results followed the guidelines of the Clinical and Laboratory Standards Institute (CLSI) veterinary breakpoints. When veterinary breakpoints were not available, human breakpoints were adopted (28). To assess whether the microorganism was MDR or non-MDR, the categorization proposed in the literature was adopted (29).

### Bacterial inoculum

Minimal inhibitory concentration (MIC) assays were performed on both field and reference bacterial strains. The bacterial inoculum was prepared according to the CLSI standard method (30). All microbiological assays were performed within 30 min after inoculum standardization. Briefly, for each strain, five bacterial colonies from fresh solid cultures were inoculated in sterile tubes with Müeller Hinton broth (MHB) and incubated at 37°C under aerobic conditions for 24 h for staphylococci, while streptococci and enterococci were incubated under microaerophilic conditions. After incubation, the bacterial suspension was centrifuged at 2000 rpm for 20 min at 4°C to separate the pellet containing bacteria from the supernatant. The pellet was subsequently resuspended in 10 mM phosphate buffer (PB), pH 7, to obtain an optical density (OD) of 0.08–0.13 at 600 nm in a 1 cm light path cuvette, corresponding to approximately 0.5 McFarland suspension (10^8^ CFU/mL). This suspension was further diluted 1:100 in sterile MHB. Then, 50 μL of the bacterial suspension, containing 10^6^ CFU/mL, was inoculated into each well to obtain a final concentration of 5×10^5^ CFU/mL. The bacterial suspensions were assessed via a Biophotometer plus (Eppendorf, Hamburg, Germany) spectrophotometer at 600 nm.

### MIC assay of plant extracts

The MIC assay was performed following the methods outlined in the CLSI standard methods, with minor modifications. Briefly, EGL-L, AA and UA were prepared as stock solutions in DMSO at concentrations of 200 mg/mL for EGL-L and 25.6 mg/mL for UA and AA. Serial twofold dilutions of each compound in DMSO were performed in a 96-well microtiter plate (Greiner, Milan, Italy). Then, one microliter of each diluted compound was added to the wells of the plates, followed by the addition of 50 μL of bacterial suspension containing 10^6^ CFU/mL, resulting in a final bacterial concentration of 5×10^5^ CFU/ml. The final dilution range for EGL-L was 2000 to 3.9 μg/mL, whereas for AA and UA, it ranged from 256 to 0.5 μg/mL. Growth and sterility controls were included for each bacterial strain and compound tested. The plates were incubated at 37°C for 24 h as described above for each bacterial strain. For each assay, three experiments were performed, with three replicates each. After incubation, the MIC was determined by visual inspection. The minimal inhibitory concentration was considered the lowest concentration able to completely inhibit bacterial growth. Furthermore, the MIC_50_ and MIC_90_ were calculated: given a graded series of MICs starting with the lowest value, MIC_50_ is the MIC value at which 50% of the isolates in a test population are inhibited (equivalent to the median MIC value), and the MIC_90_ is calculated as *n* × 0.9, where *n* represents the test strains and represents the 90th percentile, as specified in the literature (31).

### Bactericidal kinetic curves (BKC) evaluation

The bactericidal kinetic curves (BKC) of EGL-L, AA and UA were determined following the methods reported in literature (32). For each compound, three concentrations were selected based on their MIC values against the reference bacteria: 1 × MIC, 2 × MIC, and 4 × MIC. A growth control was also included. Each compound was subjected to serial twofold dilutions in DMSO in a 96-well microtiter plate (Greiner, Milan, Italy). Subsequently, 1 μL of each diluted solution was transferred to the respective wells, followed by the addition of 50 μL of bacterial suspension at a concentration of 10^6^ CFU/mL, resulting in a final bacterial concentration of 5 × 10^5^ CFU/mL. BKC were monitored by measuring the optical density at 620 nm using a spectrophotometer reading plates (Biophotometer Plus, Eppendorf, Hamburg, Germany). Measurements were taken at the following time points (T): 0, 0.5, 1, 2, 3, 4, 5, 6, 7, 8, 24, and 48 h.

### Biofilm formation assay

The biofilm-forming ability of the reference and field strains was evaluated via methods described in the literature (33), with slight modifications. The bacterial strains were cultivated in MHB at 37°C for 24 h. After incubation, the bacterial suspension was centrifuged at 2000 rpm for 20 min at 4°C, and the supernatant was removed. The pellet was resuspended in 10 mM phosphate buffer (PB), pH 7, and adjusted spectrophotometrically to obtain a final concentration of 10^7^ CFU/mL. Each well of a 96-well flat-bottomed microtiter plate was filled with 180 μL of tryptic soy broth (TSB) supplemented with 1% glucose. Thereafter, 20 μL of the previously prepared bacterial suspension was added to each well, and the plates were incubated at 37°C for 24 h. The sterility control consisted of 180 μL of TSB supplemented with 1% glucose and 20 μL of PB. Three replicates were carried out for each bacterial strain. The tests were repeated three times for each bacterial strain. After incubation, the wells were emptied and washed three times with sterile phosphate-buffered saline (PBS; pH 7.2). The residual liquid was removed by gentle flicking, and the remaining biofilm was fixed with 150 μL of methanol for 15 min. After the methanol was removed, the plates were air-dried for 2 h at room temperature. The biofilms were stained with 0.1% Hucker crystal violet (150 μL for each well) for 30 min. After staining, the crystal violet solution was removed by sequential washing with sterile deionized water. The wells were then air-dried, and 150 μL of 95% ethanol was added to each well for 30 min to solubilize the stain.

The solubilized stain was transferred to a new flat-bottomed microtiter plate, and the OD was measured at 620 nm. The biofilm-forming ability was classified on the basis of the criteria described in the literature (33, 34). Briefly, the cutoff OD (ODc) was defined as three times the standard deviation above the mean OD of the negative control, and the strains were classified as follows:

$\mathrm{OD}\leq {\mathrm{OD}}_{c}\;\text{nonadherent}\;(\mathrm{NA})$

${\mathrm{OD}}_{c}<\mathrm{OD}\leq 2\;X\;{\mathrm{OD}}_{c}\;\text{weakly adherent}\;(\mathrm{WA})$

$2\;X\;{\mathrm{OD}}_{c}<\mathrm{OD}\leq 4\;X\;{\mathrm{OD}}_{c}\;\text{moderately adherent}\;(\mathrm{MA})$

$4\;x\;{\mathrm{OD}}_{c}\leq \mathrm{OD}\;\text{strongly adherent}\;(\mathrm{StA})$ .

### Minimal inhibitory biofilm concentration assay

The minimal biofilm inhibitory concentrations (MBICs) of all the tested compounds were determined for reference and field strains classified as MA or StA. Each well of a 96-well flat-bottomed microtiter plate was filled with 178 μL of TSB supplemented with 1% glucose. Twofold dilutions of each stock solution of EGL-L, AA and UA in DMSO were prepared in a separate microtiter plate, and 2 μL of each dilution was then added to the respective wells (Greiner, Milan, Italy). The final dilution range for EGL-L was 2000 to 3.9 μg/mL, whereas for AA and UA, it ranged from 256 to 0.5 μg/mL.

The bacterial pellet was prepared as described above, resuspended in PB and adjusted spectrophotometrically to obtain a final concentration of 10^7^ CFU/mL. Thereafter, 20 μL of the bacterial suspension was added to each well. The growth control consisted of 180 μL of TSB supplemented with 1% glucose and 20 μL of bacterial suspension (10^7^ CFU/mL). The sterility control was prepared with 200 μL of TSB with 1% glucose. The plates were incubated at 37°C for 24 h. Three replicates were carried out for each bacterial strain. After incubation, biofilm staining was performed as described above.

For each tested compound and concentration, the percent inhibition was calculated via the following formula:

$${\mathrm{OD}}_{\text{inhibition}}=(1-{\mathrm{OD}}_{\mathrm{GC}}/{\mathrm{OD}}_{x})\%$$

where:

${\mathrm{OD}}_{\mathrm{GC}}=\text{Mean}\;\mathrm{OD}\;\text{of the growth control}$ .

${\mathrm{OD}}_{x}=\text{Mean}\;\mathrm{OD}\;\text{of the tested concentration}$ .

The minimal biofilm inhibitory concentration 50 (MBIC_50_) and minimal biofilm inhibitory concentration 80 (MBIC_80_) values were calculated for each bacterial strain.

The MBIC_50_ and MBIC_80_ were defined as the minimal concentrations of compounds able to inhibit 50 and 80% of biofilm formation, respectively, compared with the negative control (31).

### Statistical analysis

The data were analyzed via SPSS v29.1 software (IBM SPSS Statistics for Windows, Version 29.1; IBM Corp., Armonk, NY, USA). The variables (MICs) were checked for a normal distribution via the Kolmogorov–Smirnov normality test. Differences in MICs were subsequently assessed via the Kruskal–Wallis test. *Post hoc* analysis was performed to compare bacterial groups via pairwise comparisons of mean ranks, with Bonferroni correction.

## Results

### Chemical composition of the plant extracts

**Figure 1:**
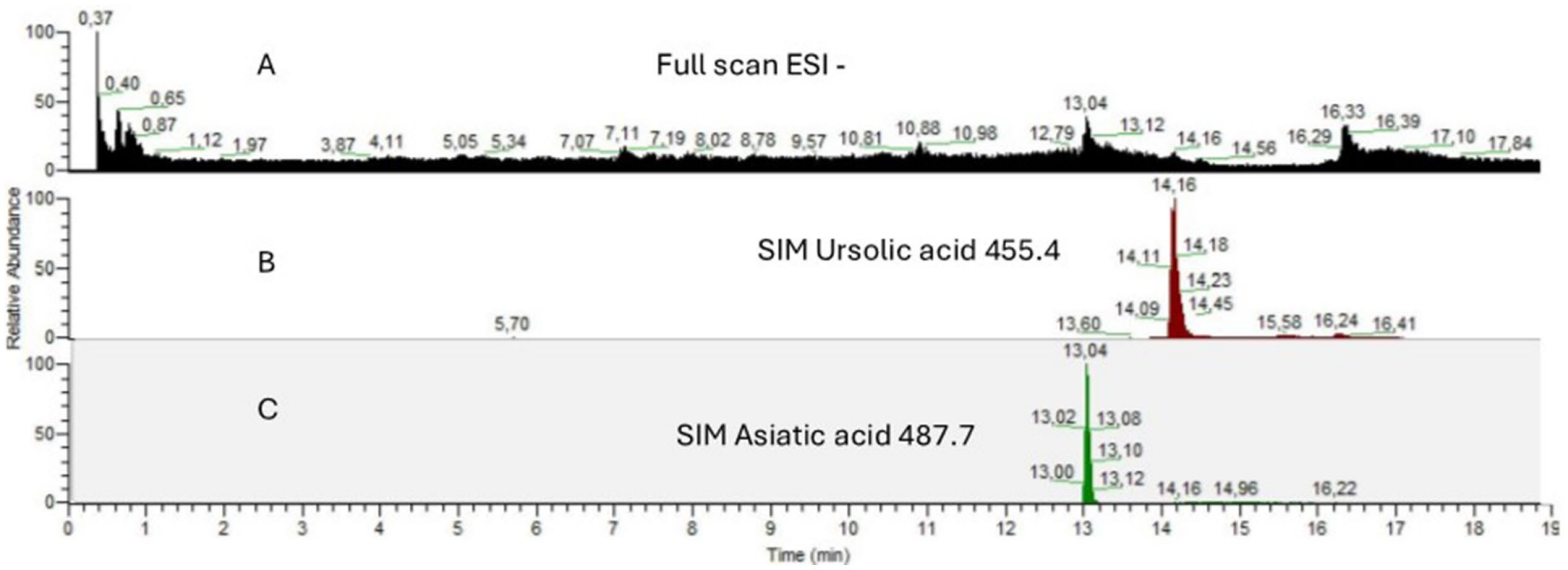
HPLC-MS chromatograms of **(A)** Full Scan in ESI negative mode of EGL-L **(B)** SIM of [M-H]^−^ m/z 455.4 for ursolic acid **(C)** SIM of [M-H]^−^ m/z 487.7 for asiatic acid.

The quantification of UA and AA in EGL-L was conducted using HPLC-MS in SIM mode. The analyses were performed in triplicate to ensure accuracy and reproducibility. Calibration curves were constructed for both compounds (calibration range: UA from 3 to 15 μg/g; AA from 5 to 20 μg/g) and subsequently used to determine their concentrations in the extract samples (calibration equation: UA *y* = 175.55x + 3002.1, *R*^2^ = 0.97; AA *y* = 1157.5x + 35,920, *R*^2^ = 0.98). Results revealed that in 1 g of EGL-L, the concentration of UA was 16.12 ± 0.32 μg/g, while AA was present at 47.29 ± 0.13 μg/g. Chromatograms of EGL-L and the two compounds under study are shown in Figure 1. The chemical composition of *Eucalyptus globulus* Labill., particularly its ethanolic extract and volatile fraction, is well-documented in the literature. Previous studies have reported a high yield of triterpenes in ethanolic and methanolic extracts, especially oleanolic acid, betulin, and betulonic acid, along with their acetylated derivatives (35). Additionally, LC-MS analysis of ethanol-extracted *E. globulus* leaves has identified antioxidant and bioactive phytochemicals, including salicylic acid β-D-glucuronide, chlorogenic acid, epicatechin, 2″-O-galloylhyperin, isoquercitrin, isorhapontin, quercitrin, and quercetin-3-O-glucuronide (36). Given the extensive literature on *E. globulus* composition, we opted for a targeted approach, focusing solely on the identification and quantification of ursolic acid and asiatic acid. These two compounds were selected due to their well-documented biological activity and were also used as pure standards in subsequent experimental studies (22).

### Antimicrobial susceptibility testing (AST)

The AST results are reported in Supplementary Tables S1–S3. The highest prevalence of MDR strains was observed in the SU group, with 14 out of 18 strains. Within this group, imipenem was the only antibiotic active against all the strains, whereas trimethoprim/sulphamethoxazole was ineffective in all the strains. In the SAG group, none of the isolates were classified as MDR. All the SAG isolates were susceptible to cefquinome, cefuroxime, imipenem, and penicillin G but resistant to trimethoprim/sulphamethoxazole. In the EN group, only one strain was identified as MDR. This strain was susceptible to amoxicillin/clavulanic acid, florfenicol, imipenem, penicillin G and vancomycin. Notably, many EN-resistant strains resistant to ampicillin (8 out of 13) were detected. The SA group presented a greater number of MDR strains than non-MDR (10 vs. 6) strains. In particular, 3 out of 15 strains were resistant to cefoxitin, a phenotypic marker for MRSA. On the other hand, cefquinome, fusidic acid, florfenicol, imipenem and rifaximin were the most active antibiotics in this group of bacteria, with 14 out of 15 strains being susceptible to these antibiotics.

### MICs of EGL, UA, and AA against reference strains

As reported in Table 1, all the compounds involved in this study showed antimicrobial activity against the reference bacterial strains. EGL-L showed the highest antimicrobial activity against SA ATCC 25923 and SU ATCC 19496, with an MIC of 250 ± 0 μg/mL, while its lowest activity was observed against SAG ATCC 27956, with an MIC of 500 ± 0 μg/mL. Ursolic acid had the lowest MIC value against SU ATCC 19496 (4 ± 0 μg/mL) and the highest MIC values against SAG ATCC 27956 and MRSA ATCC 43300, both at 24 ± 0 μg/mL. Finally, AA had the highest activity against SU ATCC 19496, with an MIC of 4 ± 0 μg/mL, and the lowest activity against MRSA ATCC 43300 and SA ATCC 25923, with an MIC of 32 ± 0 μg/mL.

**Table 1:** Minimal Inhibitory Concentrations (MICs) of *Eucalyptus globulus* leaves extract (EGL-L), ursolic (UA) and asiatic acids (AA) against reference bacterial strains.

Reference strains	Compounds	MIC (μg/mL)	SD
*Streptococcus agalactiae* ATCC 27956	EGL-L	500	0
	UA	24.0	11.30
	AA	8.00	0
*Streptococcus uberis* ATCC 19496	EGL-L	250	0
	UA	4.00	0
	AA	4.00	0
*Enterococcus faecium* ATCC 19434	EGL-L	375	176.8
	UA	10.0	8.50
	AA	8.00	0
MRSA ATCC 43300	EGL-L	375	176.8
	UA	24.0	11.30
	AA	32.0	0
*Staphylococcus aureus* ATCC 25923	EGL-L	250	0
	UA	12.0	5.70
	AA	32.0	0

### MIC_50_ AND MIC_90_ of EGL-L, UA, and AA against field strains

**Figure 2:**
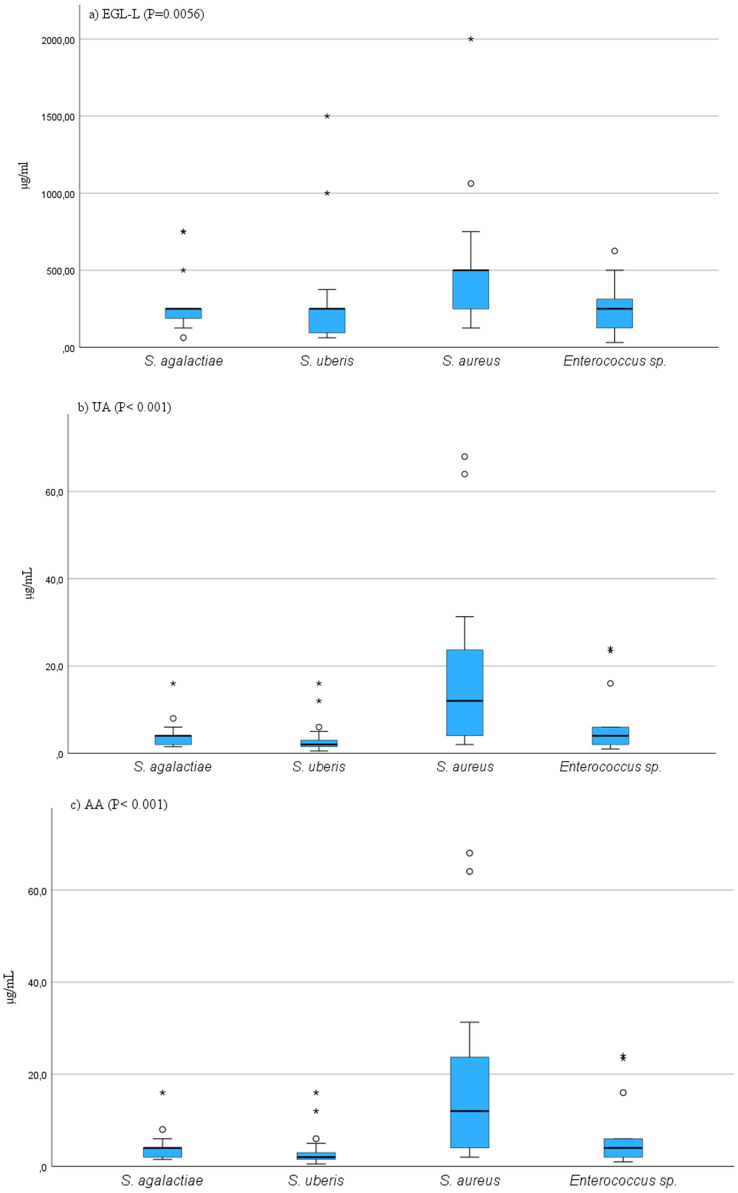
**(a–c)** Results of Kruscal-Wallis comparison among MIC values of field bacterial species for Eucalyptus globulus leaves extract (EGL-L), ursolic acid (UA) and asiatic acid (AA). On y-axis are showed the MIC values, on x-axis are showed bacterial species.

**Figure 3:**
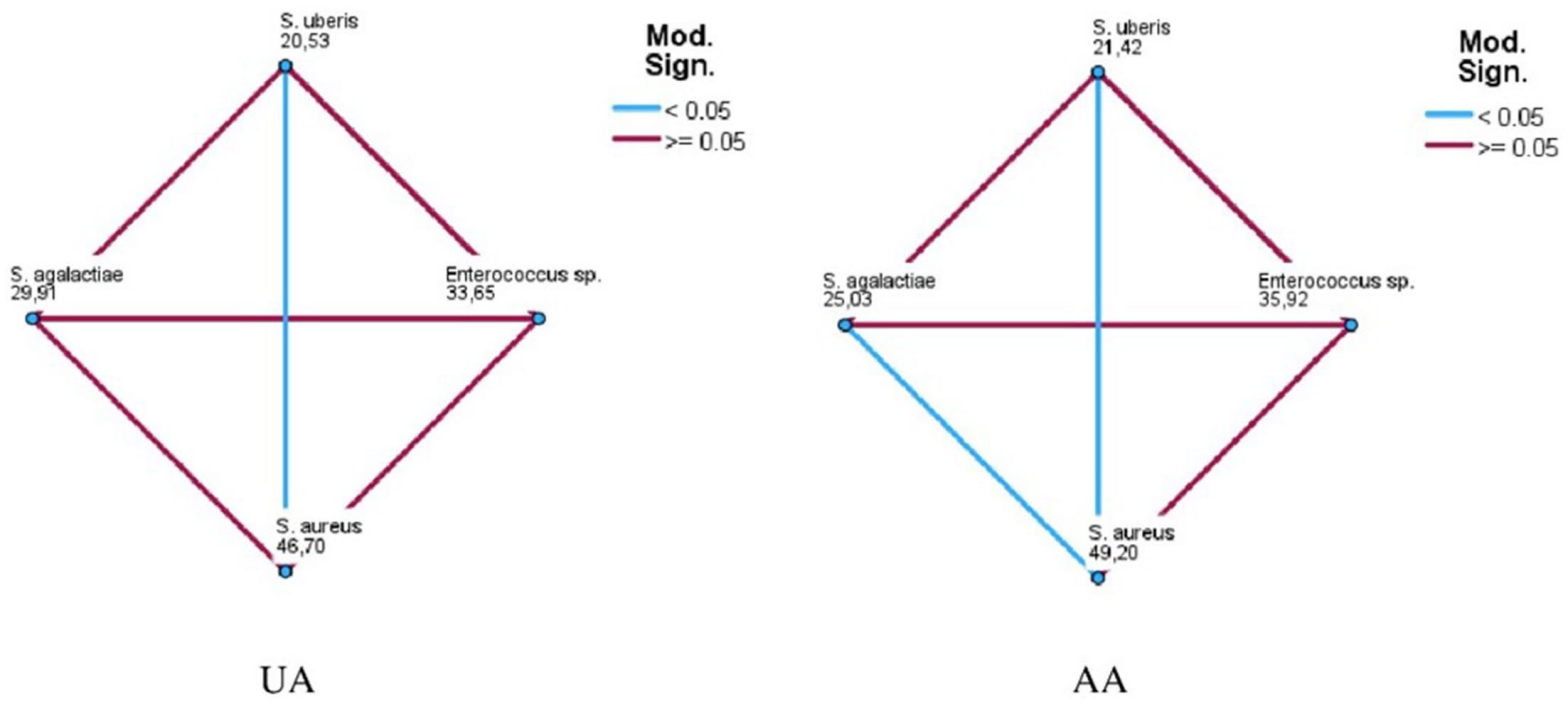
Pairwise comparison among field bacterial species for ursolic acid (UA) and asiatic acid (AA).

The MIC_50_ and MIC_90_ results for EGL-L, UA and AA are reported in Table 2. The *Eucalyptus globulus* leaf extract had the highest MIC_50_ and MIC_90_ values against SA strains (500 μg/mL and 1062.50 μg/mL, respectively). An MIC_50_ value of 250 μg/mL was obtained for EGL-L against all the other bacterial groups, while the lowest MIC_90_ (500 μg/mL) was observed against the EN group. The lowest MIC_50_ and MIC_90_ values for UA were observed in the SU group (2.0 μg/mL) and SAG group (8 μg/mL), respectively. UA had the highest MIC_50_ and MIC_90_ values against the SA group (12 and 64 μg/mL, respectively). The lowest MIC_50_ and MIC_90_ values for AA were detected in the SU and SAG groups (6 and 16 μg/mL, respectively), whereas the highest MIC_50_ and MIC_90_ values were detected in the SA group (24 μg/mL and 187.50 μg/mL, respectively). Overall, the MIC_50_ and MIC_90_ values for EGL-L were significantly greater than those observed for UA and AA (*p* < 0.001). Moreover, the Kruskal–Wallis nonparametric test revealed significant differences in the median MIC values between the different bacterial groups for UA (<0.001) and AA (<0.001), whereas no significant difference was detected for EGL-L (*p* = 0.056) (Figures 2a–c). The pairwise comparison among each bacterial group revealed that for UA, the SA group was significantly different from the SU group (*p* < 0.05). For AA, the pairwise comparison revealed a significant difference between the SA group and the SU/SAG group (p < 0.05) (Figure 3). In Table 3 are showed the MIC average of EGL-L, UA and AA against MDR field strains of *S. aureus* and *S. uberis*.

**Table 2:** Minimal Inhibitory Concentration on 50% of the strains (MIC_50_) and Minimal Inhibitory Concentration on 90% of the strains (MIC_90_) of *Eucalyptus globulus* leaves extract (EGL-L), ursolic (UA) and asiatic acids (AA) against field strains isolated from cows with mastitis.

Bacterial species	Compounds	MIC_50_ (μg/mL)	MIC_90_ (μg/mL)
*Streptococcus agalactiae*	EGL-L	250	750
	UA	4.0	8.00
	AA	8.0	16.0
*Streptococcus uberis*	EGL-L	250	1000
	UA	2.0	12.0
	AA	6.0	23.5
*Staphylococcus aureus*	EGL-L	500	1062.5
	UA	12.0	64.00
	AA	24.0	187.5
*Enterococcus* sp.	EGL-L	250.0	500
	UA	4.00	23.4
	AA	16.0	32.0

**Table 3:** Minimal Inhibitory Concentrations (MICs) of *Eucalyptus globulus* leaves extract (EGL-L), ursolic (UA) and asiatic acids (AA) against MDR field strains isolated from cows with mastitis.

Bacterial strains – MDR	EGL-L	UA	AA
	MIC (μg/mL) ± SD		
*S. aureus* MDR	618.8 ± 559.4	21.6 ± 24.4	37.2 ± 53.5
*S. uberis* MDR	257.2 ± 242.1	3.10 ± 4.09	15.7 ± 35.2

### Bactericidal kinetic curves (BKC) against reference strains

**Figure 4:**
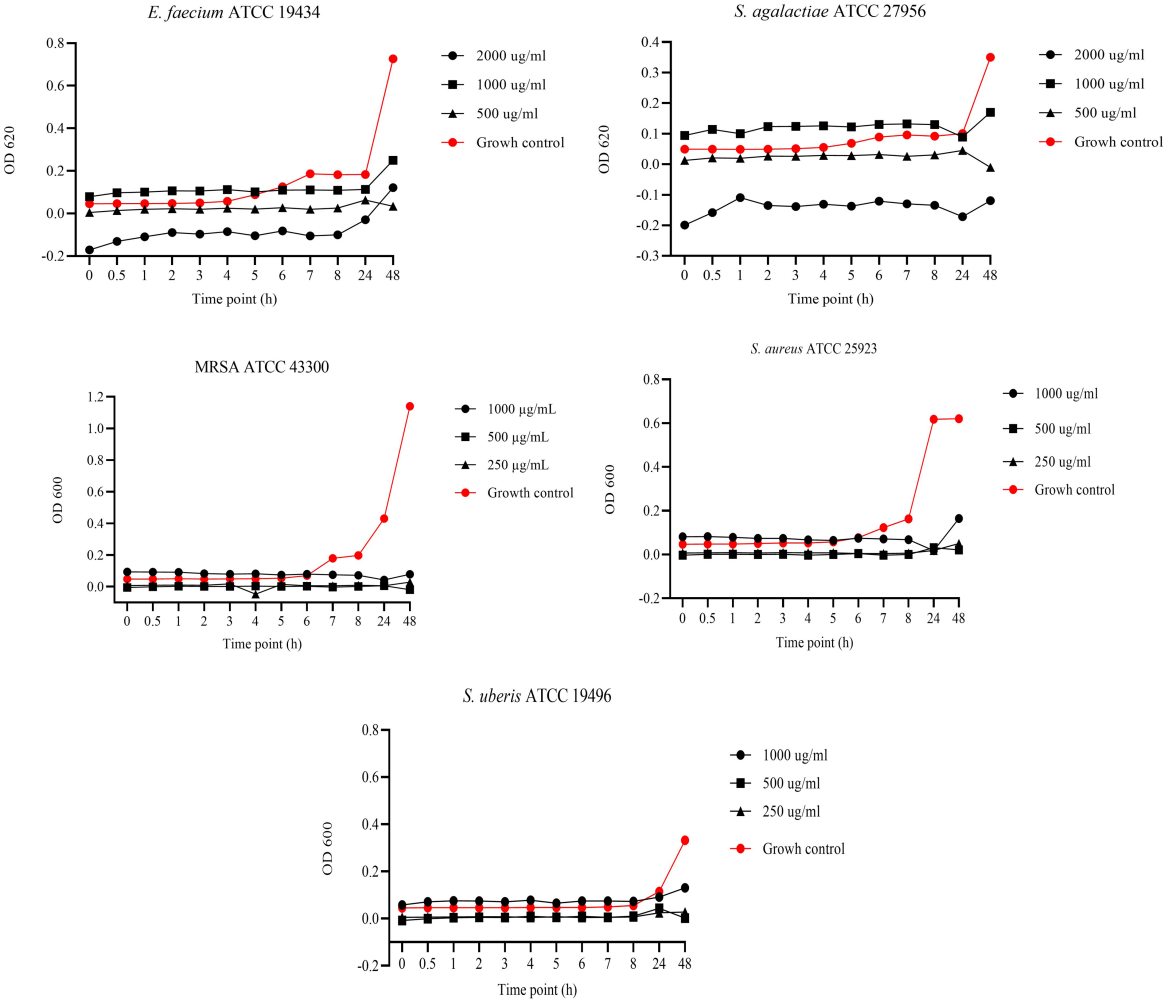
Bactericidal Kinetic Curves (BKC) of *Eucalyptus globulus* Leaves Extract (EGL-L) against tested reference bacteria. On *y* axis are reported the OD values of bacterial growth, on *x* axis the timepoint of incubation.

Figure 4 presents the results of BKC of EGL-L against reference bacterial strains. For *E. faecium* and *S. agalactiae*, the tested concentrations were 2000, 1,000, and 500 μg/mL, while for MRSA, *S. aureus*, and *S. uberis*, the concentrations tested were 1,000, 500, and 250 μg/mL. For *E. faecium,* all tested concentrations showed a bacteriostatic effect; however, the 2000 μg/mL concentration demonstrated the highest antimicrobial activity at all experimental time points. This concentration exhibited similar results against *S. agalactiae*. In contrast, for MRSA and *S. aureus*, all tested concentrations showed similar OD values to the growth control up to 6 h. However, during the logarithmic growth phase, a strong antimicrobial activity was observed, inhibiting bacterial growth at the remaining time points. For *S. uberis*, the 500 and 250 μg/mL concentrations showed lower OD values than the growth control throughout the experiment, while the 1,000 μg/mL concentration only exhibited this effect at 24 and 48 h.

**Figure 5:**
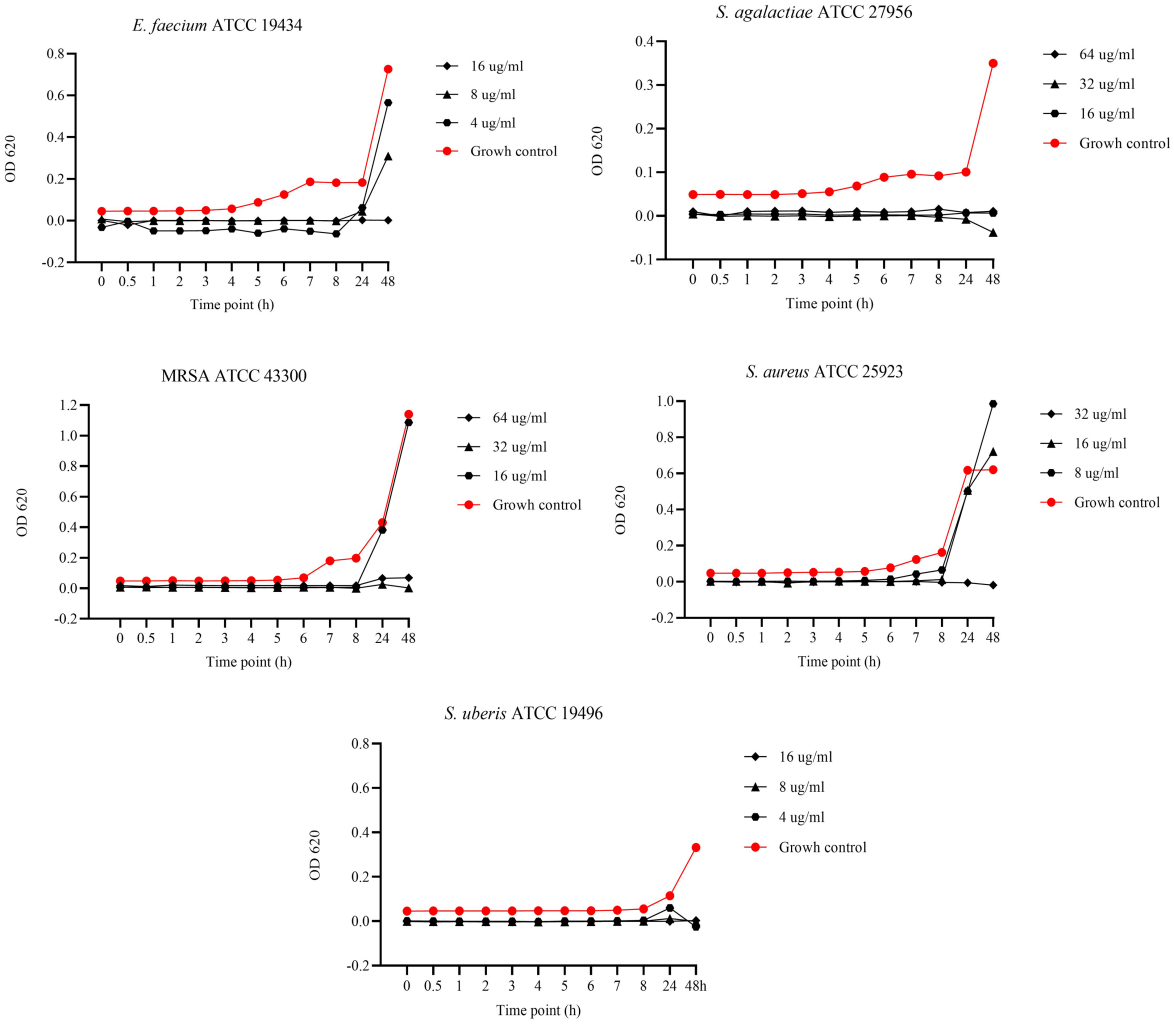
Bactericidal Kinetic Curves (BKC) of ursolic acid (UA) against tested reference bacteria. On *y* axis are reported the OD values of bacterial growth, on *x* axis the timepoint of incubation.

Figure 5 shows the results of the BKC of UA against reference bacterial strains. Specifically, concentrations of 64, 32, and 16 μg/mL were tested against *S. agalactiae* and MRSA, while 32, 16, and 8 μg/mL were tested against *S. aureus*. Finally, concentrations of 16, 8, and 4 μg/mL were tested against *S. uberis* and *E. faecium*. Regarding *S. agalactiae*, all tested concentrations demonstrated bactericidal activity. In contrast, against MRSA, bactericidal effects were observed only at 64 and 32 μg/mL, while 16 μg/mL exhibited bacteriostatic activity up to 8 h. After the logarithmic phase, at 24 h, the OD values of the 16 μg/mL concentration were similar to those of the growth control, indicating bacterial growth. A similar pattern was observed against *S. aureus*, with only 32 μg/mL exhibiting bactericidal activity. In contrast, concentrations of 16 and 8 μg/mL demonstrated bacteriostatic effects up to 8 h, prior to the logarithmic phase. For *E. faecium*, 8 and 4 μg/mL concentrations exhibited bacteriostatic effects up to 24 h, while the 16 μg/mL concentration showed a bactericidal effect. Finally, against *S. uberis*, the 16 and 8 μg/mL concentrations showed bactericidal effects.

**Figure 6:**
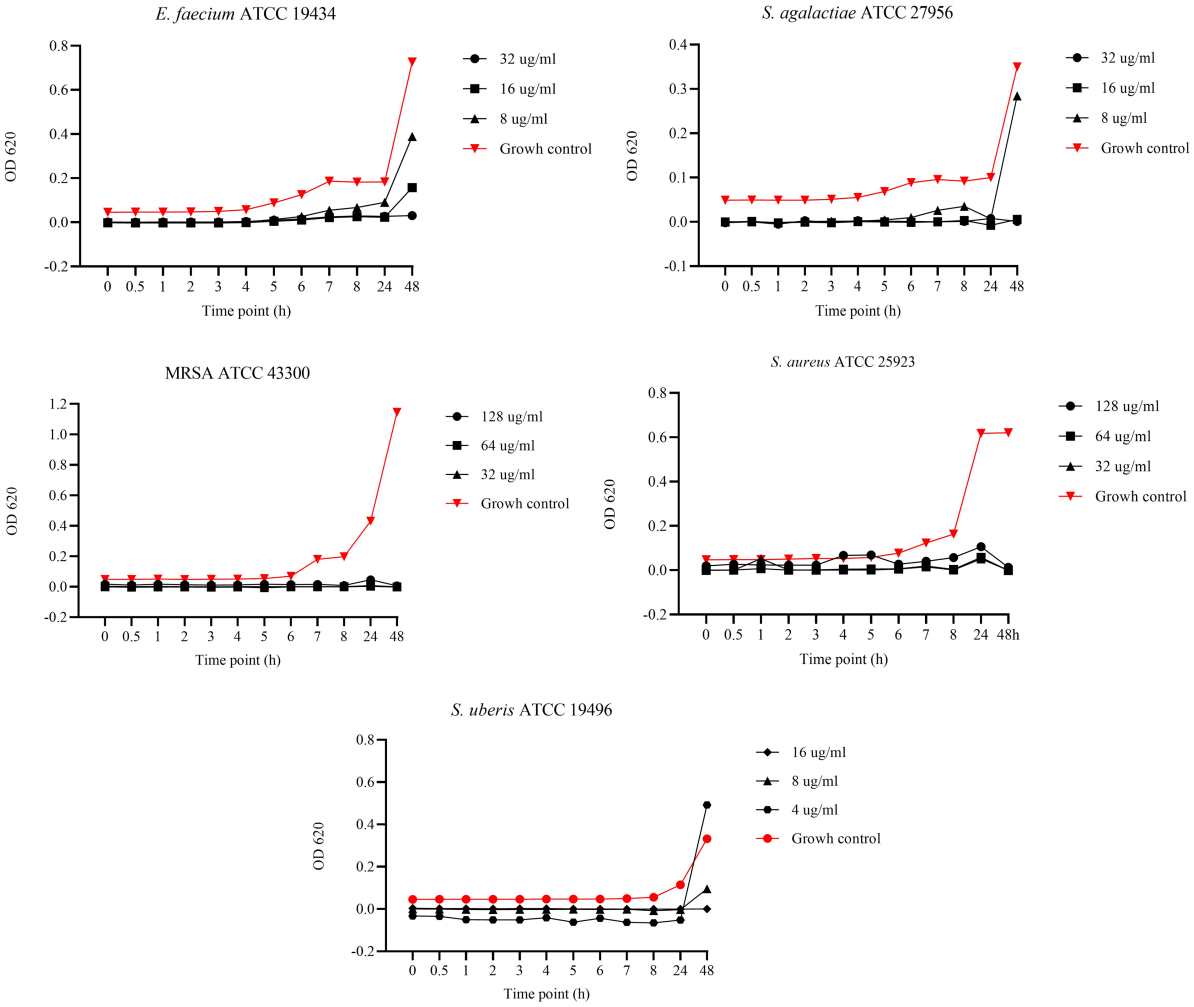
Bactericidal Kinetic Curves (BKC) of asiatic acid (AA) against tested reference bacteria. On *y* axis are reported the OD values of bacterial growth, on *x* axis the timepoint of incubation.

Figure 6 displays the results of the BKC of AA against reference strains. Specifically, concentrations of 128, 64, and 32 μg/mL were tested against MRSA and *S. aureus*, while 32, 16, and 8 μg/mL were tested against *E. faecium* and *S. agalactiae*. Lastly, concentrations of 16, 8, and 4 μg/mL were tested against *S. uberis*. Asiatic acid exhibited strong antimicrobial activity with bactericidal effect against *MRSA* and *S. aureus* at all tested concentrations. In contrast, against *E. faecium* and *S. agalactiae*, bactericidal effect was observed only at 32 and 16 μg/mL, while 8 μg/mL showed a bacteriostatic effect up to 24 h. A similar pattern was observed against *S. uberis*, where 16 and 8 μg/mL showed bactericidal effects, while the 4 μg/mL concentration exhibited a bacteriostatic effect up to 24 h.

### Biofilm formation of reference and field strains

The evaluation of biofilm formation by reference strains revealed that only SA ATCC 25923 was moderately adherent, whereas MRSA ATCC 43300 was strongly adherent. All the other reference strains exhibited weak adherence. For the field strains, nonbiofilm producers (nonadherent) belonged to the EN (5 out of 13; 38.5%) and SU (8 out of 18; 44.4%) groups. Weak biofilm producers were most common in the SU group (8 out of 18; 44.4%) and EN group (7 out of 13; 53.8%) but least common in the SA group (1 out of 15; 6.7%). Conversely, the SA group presented the highest proportion of moderate biofilm-producing strains (6 out of 15; 40.0%), followed by the SAG group (5 out of 17; 29.5%), SU group (2 out of 18; 11.1%) and EN group (1 out of 13; 7.7%) (Table 4).

**Table 4:** Minimal Biofilm Inhibitory Concentrations 50 and 80 (MBIC_50_ and MBIC_80_) of *Eucalyptus globulus* leaves extract (EGL-L), ursolic (UA), and asiatic acid (AA) against moderately adherent reference and field strains.

Moderately adherent bacteria	MBIC_50_ (μg/mL)			MBIC_80_ (μg/mL)		
	EGL-L	UA	AA	EGL-L	UA	AA
*S. aureus* ATCC 25923	3.96	32.00	16.00	7.81	32.0	16.0
*S. uberis* BV2	>2000	256	8.00	>2000	>256	>256
*S. uberis* BV16	125	4.00	4.00	>2000	>256	>256
*S. agalactiae* IZS4	31.25	128	4.00	1000	128	32.0
*S. agalactiae* IZS5	2000	0.50	2.00	>2000	8.00	32.0
*S. agalactiae* IZS10	500	4.00	2.00	>2000	8.00	4.00
*S. agalactiae* IZS11	250	0.50	4.00	>2000	8.00	8.00
*S. agalactiae* IZS12	2000	8.00	2.00	>2000	8.00	4.00
*Enterococcus* sp. BV21	7.81	65.5	4.00	>2000	256	>256
*S. aureus* BV16	125	1.00	< 0.50	125	32.0	32
*S. aureus* IZS4	15.63	16.0	16.0	62.5	32.0	16.0
*S. aureus* IZS5	125	16.0	16.0	>2000	>256	>256
*S. aureus* IZS7	15.63	32.0	16.0	>2000	256	16.0
*S. aureus* IZS8	62.50	16.0	16.0	62.5	16.0	16.0
*S. aureus* IZS9	31.25	16.0	16.0	125	16.0	16.0

Finally, strong biofilm-producing bacteria were found only in the SA group (8 out of 15; 53.3%) and SAG group (8 out of 17; 47.0%).

### MBIC_50_ and MBIC_80_ of EGL, UA, and AA on moderately adherent (MA) field and reference strains

The MBIC_50_ and MBIC_80_ results are reported in Table 4.

For *Staphylococcus aureus* ATCC 25923, EGL-L had an MBIC_50_ and MBIC_80_ of 3.96 μg/mL and 7.81 μg/mL, respectively. The UA MBIC_50_ and MBIC_80_ values were identical (32 μg/mL). The MBIC_50_ and MBIC_80_ for AA were both 16 μg/mL. With respect to EGL-L, an MBIC_50_ was found for all the field MA strains, except for *S. uberis* BV2 (>2000 μg/mL). The lowest MBIC_50_ value was observed for *Enterococcus* sp. BV21 (7.81 μg/mL), while the highest value was found for *S. agalactiae* IZS5 and IZS12 (2000 μg/mL). Most of the MA field strains reported an MBIC_80_ > 2000 μg/mL for EGL-L (9 out of 14).

Ursolic acid had the lowest MBIC_50_ for *S. agalactiae* IZS5 and IZS11 (0.50 μg/mL), while the highest value was observed for *S. uberis* BV2 (256 μg/mL). An MBIC*_80_* of 8 μg/mL was obtained for UA on all SAG field strains identified as moderately adherent, except for *S. agalactiae* IZS4 (128 μg/mL), whereas an MBIC_80_ > 256 μg/mL was reported for *S. uberis* BV2, BV16 and *S. aureus* IZS5.

Finally, AA had the lowest MBIC_50_ for *S. aureus* BV16 (< 0.5 μg/mL) and the lowest MBIC*_80_* for *S. agalactiae* IZS10 (4 μg/mL).

### MBIC_50_ and MBIC_80_ of EGL, UA, and AA against strongly adherent reference and field strains

The results of the MBIC_50_ and MBIC_80_ values for the StA reference and field strains are reported in Table 5. For MRSA ATCC 43300, the MBIC_50_ and MBIC_80_ values of EGL-L were 125 μg/mL and 250 μg/mL, respectively. For UA, the MBIC_50_ and MBIC_80_ were 32 μg/mL and 64 μg/mL, respectively. For AA, both the MBIC_50_ and the MBIC_80_ were 16 μg/mL. For the field strains, the lowest MBIC_50_ for EGL-L was observed for *S. aureus* BV44 (3.9 μg/mL), whereas the highest was observed for *S. agalactiae* IZS13 (500 μg/mL). Notably, 8 out of 16 StA strains presented an MBIC_80_ > 2000 μg/mL. Ursolic acid had the lowest MBIC_50_ (0.5 μg/mL) against *S. aureus* BV44, *S. agalactiae* IZS2 and IZS6, whereas *S. aureus* BV5 and *S. agalactiae* IZS7 presented MBIC_80_ values > 256 μg/mL.

**Table 5:** Minimal biofilm inhibitory concentration (MBIC_50_ and MBIC_80_) of *Eucalyptus globulus* leaves extract (EGL-L), ursolic (UA) and asiatic acid (AA) against strongly adherent reference and field strains.

Strongly adherent bacteria	MBIC_50_ (μg/mL)			MBIC_80_ (μg/mL)		
	EGL-L	UA	AA	EGL-L	UA	AA
MRSA ATCC 43300	125	32.0	16.0	250	64.0	16.0
*S. aureus* BV5	31.25	16.0	4.00	62.5	>256	8.00
*S. aureus* BV30	250	32.0	8.00	500	32.0	8.00
*S. aureus* BV42	7.81	4.00	4.00	7.81	0.50	4.00
*S. aureus* BV43	31.25	4.00	2.00	>2000	256	4.00
*S. aureus* BV44	3.90	0.50	2.00	62.5	8.00	4.00
*S. aureus* IZS2	125	32.0	16.0	>2000	256	16.0
*S. aureus* IZS3	62.5	32.0	16.0	>2000	64.0	16.0
*S. aureus* IZS6	62.5	64.0	16.0	62.5	64.0	16.0
*S. agalactiae* IZS2	31.25	0.50	128	500	0.50	128
*S. agalactiae* IZS3	125	4.00	32	125	256	32.0
*S. agalactiae* IZS6	125	0.50	2.00	>2000	8.00	32.0
*S. agalactiae* IZS7	15.63	1.00	4.00	>2000	>256	>256
*S. agalactiae* IZS8	31.25	1.00	2.00	125	2.00	2.00
*S. agalactiae* IZS13	500	16.0	8.00	>2000	16.0	8.00
*S. agalactiae* IZS14	7.81	4.00	8.00	>2000	4.00	8.00
*S. agalactiae* IZS15	62.5	4.00	8.00	>2000	8.00	8.00

For AA, the lowest MBIC_50_ value (2 μg/mL) was observed for *S. aureus* BV43 and BV44 and *S. agalactiae* IZS6 and IZS8; the latter also presented the lowest MBIC_80_ at the same value. The highest MBIC_50_ was observed for *S. agalactiae* IZS2 (128 μg/mL). Finally, the highest MBIC_80_ for AA was observed for *S. agalactiae* IZS7 (>256 μg/mL).

## Discussion

Antibiotics represent the reference treatment for BM, with penicillins, aminoglycosides and tetracyclines being the most commonly used classes (7). This study assessed the susceptibility of several bacterial strains not only to antibiotics commonly used to treat BM but also to molecules classified as critically important for human health and banned in veterinary use (11). Some commonly used antibiotics, such as ampicillin, gentamicin and trimethoprim/sulphamethoxazole, were ineffective against most of the clinical strains considered. In contrast, third-generation cephalosporins and imipenem are the most active antibiotics, although their use is restricted in veterinary clinical practice, as they belong to EMA categories A and B (37). Thirty-nine percent of the isolated strains, mainly *S. uberis* and *S. aureus*, were classified as MDROs according to the classification proposed in the literature (29). Of particular concern were *S. uberis* isolates resistant to penicillin, cephalosporins and, in one case, vancomycin. This finding was in agreement with the results reported by other authors (38). The emergence of MDROs in veterinary medicine is of concern, as it may result in a reduction in therapeutic options for patients (39). For this reason, in recent years, researchers have focused on alternative therapeutic approaches to conventional antimicrobial agents (4). Among them, plant extracts and essential oils have shown interesting antimicrobial activity against mastitis pathogens (12). This study investigated a plant extract of *Eucalyptus globulus* leaves from Rwanda and its main pentacyclic triterpenes, AA and UA, which are well documented for their antimicrobial activity (16, 40, 41). The comparison of the results of UA and AA quantification in EGL-L and their antimicrobial activity indicates that the activity of EGL-L, even at the highest tested concentration (2000 μg/mL), is not solely due to the presence of AA and UA, given that these are present at concentrations significantly lower than their MIC values. Rather, it is also due to the synergistic activity of the phytocomplex, which is particularly rich in ursane, oleanane and lupane skeletons. Even if the MIC values of EGL-L were higher than those of AA and UA, there were no significant differences among the bacterial populations, indicating the same efficacy in all the considered bacterial groups. Conversely, for both AA and UA, different efficacy were observed among the bacterial groups. In particular, UA had significantly lower MIC values in *S. uberis* than in *S. aureus*. For AA, the same significant difference was observed between the two *Streptococcus* groups and *S. aureus*. All three tested compounds were effective against MDROs. Compared with those of conventional antibiotics, their efficacy may be due to the plurality of targets of the extract and the pentacyclic triterpenes, indicating the possibility of using these compounds to treat MDR-BM (9). These findings are consistent with the existing literature, which reports strong antimicrobial activity of UA against *S. aureus* ATCC 25923 (MIC = 8 μg/mL) and multidrug-resistant organisms (MDROs) such as MRSA (MIC = 3 μg/mL) and Vancomicin-resistant *Enterococcus* (VRE) (MIC = 4 μg/mL). Similarly, our results regarding AA align with previous research. One study evaluated its antimicrobial activity against foodborne bacterial pathogens isolated from contaminated chicken, duck, and dairy products, reporting strong activity against Gram-positive bacteria, with MIC values comparable to our findings (28 ± 2 μg/mL for *S. aureus* and 20 ± 2 μg/mL for *E. faecalis*). However, a direct comparison of the antimicrobial activity of AA and UA against Gram-negative bacteria is not feasible, as our study exclusively focused on Gram-positive bacteria isolated during bovine mastitis. Nonetheless, literature indicates that UA exhibits moderate to limited activity against *E. coli* (MIC = 50 μg/mL), *Salmonella Typhi* (MIC = 50 μg/mL), and *Pseudomonas aeruginosa* (MIC > 256 μg/mL). In contrast, AA has been reported to be more effective against foodborne bacterial strains (*E. coli* O157:H7, *S.* Typhimurium DT104, and *P. aeruginosa*), with MIC values below 40 μg/mL (16, 41).

In our study, we evaluated the BKC of EGL-L, AA, and UA against reference strains, testing three concentrations starting from their MIC values. The EGL-L extract predominantly exhibited a bacteriostatic effect at 2000 μg/mL against *E. faecium*, *S. agalactiae*, and *S. uberis*, while all tested concentrations (1,000, 500, and 250 μg/mL) showed this effect against MRSA and *S. aureus*. In contrast, UA exhibited both bactericidal and bacteriostatic effects depending on the concentration and bacterial strain. It was bactericidal against *S. uberis* (16, 8 and 4 μg/mL) and *S. agalactiae* (64, 32, and μg/mL), while only the highest concentrations showed bactericidal activity against *E. faecium* and *S. aureus*, respectively 16 and 32 μg/mL. Against MRSA, higher concentrations (64 and 32 μg/mL) were bactericidal, whereas 16 μg/mL exhibited bacteriostatic effect up to 8 h. Regarding AA, it displayed the strongest antimicrobial activity, with a bactericidal effect at all tested concentrations (128, 64, and 32 μg/mL) against MRSA and *S. aureus*. Against *E. faecium*, S*. agalactiae*, and *S. uberis*, bactericidal effects were only observed at highest concentrations, while the lowest concentrations (8 μg/mL for *E. faecium* and *S. agalactiae* and 4 μg/mL for *S. uberis*) exhibited bacteriostatic effects up to 24 h. These results indicate that EGL-L primarily exerts a bacteriostatic effect against all tested strains, while both pentacyclic triterpenes exhibited either bactericidal or bacteriostatic activity, depending on the concentration and the bacterial strain.

Biofilms act as a defense mechanism that enables bacteria to evade the immune response, resist conventional disinfectants, and reduce the effectiveness of antibiotic treatment (42). As a result, alternative strategies, including the use of plant-derived compounds aimed at either preventing biofilm formation or eradicating preformed biofilms, have been explored in recent years (40, 41). As reported in the literature (12), several compounds of plant origin have also been evaluated for their antibiofilm activity toward the main pathogens of BM. Several studies have assessed the activity of UA against biofilms formed by clinical isolates from bovine mastitis (BM). Notably, UA has been shown to effectively inhibit the formation of *Staphylococcus aureus* and *Streptococcus uberis* biofilms derived from BM at concentrations similar to those observed in this study. At concentrations of 30 and 100 μg/mL, UA inhibited 33.96 ± 3.17% and 57.40 ± 2.8% of *S. uberis* biofilm formation, respectively. For *S. aureus* from BM, UA demonstrated a stronger inhibitory effect, with 71.5 and 48.6% inhibition at concentrations of 60 μg/mL and 30 μg/mL, respectively (43, 44). In contrast, one study reports the antibiofilm activity of *Eucalyputs globulus* extract against biofilm produced solely by *S. aureus* from bovine with mastitis (5). The preliminary qualitative and quantitative assessment of the biofilm-producing ability of mastitis isolates revealed that most of our strains classified as moderate or strong biofilm producers were *S. aureus* or *S. agalactiae* species. This finding agrees with the literature (42–47), where *S. aureus* and *S. agalactiae* are indicated as the bacteria that produce the most biofilm bacteria involved in bovine mastitis. With respect to the antibiofilm activity of EGL-L, AA and UA, none of them were able to completely inhibit (99.9%) biofilm formation. However, partial inhibition, measured as the MBIC_50_ or MBIC_80_, was detected. The extract had an MBIC_80_ ≥ 2000 μg/mL in more than half of the moderate and strong biofilm-producing strains. However, all the considered strains (except one) presented an MBIC_50_ ≤ 2000 μg/mL. Another study reported the antibiofilm activity of *Eucalyptus globulus* extract, attributing its effects to a reduction in bacterial populations caused by inhibited microbial respiration, increased plasma membrane permeability, ion leakage, or the hydrophilic nature of the bacterial cell wall (48). The pentacyclic triterpenes were much more effective than the natural extract at inhibiting biofilms, since an MBIC_80_ was obtained for all the tested bacteria, except for five isolates. In addition, AA had the highest antibiofilm activity, with an MBIC_80_ ≤ 32 μg/mL for most of the tested strains. According to the literature (19), the stronger antibiofilm activity of AA than that of UA could be due to different chemical structures. This could allow AA to penetrate bacterial cells more effectively, hindering their adhesion and thereby preventing biofilm formation.

Analyzing the antimicrobial and antibiofilm activity of EGL-L, it is observed that the MIC₉₀ values for *S. aureus* and *S. agalactiae*, the main biofilm-producing bacteria, are higher than the concentrations required to achieve MBIC₅₀, particularly against *S. aureus*. This suggests that the extract only partially inhibits biofilm formation at these concentrations. This finding is consistent with the literature, which reports that *E. globulus* extract exhibits antibiofilm activity against *S aureus* biofilms from bovine mastitis. The author reports that concentrations 8–32 times higher than the MIC are required, supporting the evidence that biofilms are 10–1,000 times more resistant than planktonic cells (5). Regarding UA, at MIC₉₀, it achieves MBIC₅₀ for all *S. aureus* strains and all but two *S. agalactiae* strains. However, for MBIC₈₀, several strains require higher concentrations. Furthermore, AA exhibits a similar behavior to UA but it is able to achieve both MBIC₅₀ and MBIC₈₀ at MIC₉₀ for all *S. aureus* strains except one, while for *S. agalactiae*, it only reaches MBIC₅₀.

In conclusion, EGL-L only partially inhibits biofilm formation, particularly in *S. aureus*, while UA and AA demonstrate greater efficacy. UA achieves MBIC₅₀ for nearly all strains at MIC₉₀, whereas AA appears even more active against *S. aureus*. However, for both, MBIC₈₀ requires higher concentrations, indicating a dose-dependent effect. These findings highlight the antibiofilm potential of UA and AA, with AA showing particularly promising results, suggesting significant prospects for future studies.

## Conclusion

Bovine mastitis is the main cause of economic losses in dairy cattle farming because of the early culling of affected animals and the lack of economic income from wasting milk. Furthermore, the presence of MDROs on farms could reduce the number of therapeutic options available for treating affected animals.

In the present study, a plant extract derived from *Eucalyptus globulus* leaves was evaluated, along with its main active components AA and UA. All the tested compounds exhibited notable antimicrobial activity against MDROs. However, as expected, the pure compounds AA and UA showed lower MIC values on all field bacterial strains compared to EGL-L. Despite this, the extract demonstrated similar efficacy across all bacterial groups, possibly indicating a broader therapeutic potential compared to the two pentacyclic triterpenes, which exhibited more selective antimicrobial activity. Pentacyclic triterpenes, particularly AA, displayed promising antibiofilm activity, especially against strongly adherent field strains of bovine mastitis. These findings suggest that AA is the most promising alternative to conventional antimicrobials among the compounds tested. Asiatic acid has the potential to be used topically, intramammary, for the control and prevention of bovine mastitis, particularly due to its efficacy against biofilm formation. Future studies will be necessary to assess the *in vitro* cytotoxicity of these compounds, both on common cell lines used for screening new alternative compounds and on specific cell lines. Furthermore, *in vivo* studies and formulation development will be required to evaluate their effective topical use in the treatment of bovine mastitis.

## Data Availability

The raw data supporting the conclusions of this article will be made available upon request to interested researchers by the authors.
