# Supplementary material for: Antibacterial and antibiofilm activity of Eucalyptus globulus leaf extract, asiatic acid and ursolic acid against bacteria isolated from bovine mastitis
Source: Front Vet Sci. 2025 May 14;12:1565787. doi: 10.3389/fvets.2025.1565787 (PMC12117823; doi:10.3389/fvets.2025.1565787)
Supplement: Supplementary file 1 [file Table_1.docx]

**S-Table 1. Antibiotics susceptibility testing (AST) of Streptococcus spp. isolated from bovine with mastitis.**

| **Field Strains** | Antibiotics | | | | | | | | | | | | | | | |
| --- | --- | --- | --- | --- | --- | --- | --- | --- | --- | --- | --- | --- | --- | --- | --- | --- |
|  | AUG | AMP | CFQ | CXM | CZ | EFT | E | ENR | FFC | IMI | MAR | OT | PG | RAX | SXT | VA |
| *S. uberis* | S | R | S | R | S | R | R | I | R | S | R | R | S | S | R | S |
| *S. uberis* | R | R | I | R | S | R | I | R | I | S | S | R | S | S | R | S |
| *S. uberis* | S | R | S | S | S | I | I | I | I | S | I | R | S | S | R | S |
| *S. uberis* | R | R | I | S | R | I | S | I | I | S | I | R | S | S | R | I |
| *S. uberis* | S | R | S | S | S | S | R | S | S | S | S | R | S | S | R | S |
| *S. uberis* | R | R | I | R | S | I | I | I | I | S | I | R | R | I | R | S |
| *S. uberis* | S | R | I | R | R | I | S | I | S | S | S | R | S | S | R | S |
| *S. uberis* | R | R | I | S | S | S | S | I | S | S | I | R | S | S | I | S |
| *S. uberis* | R | R | S | S | S | S | R | I | I | S | S | R | S | S | R | S |
| *S. uberis* | S | R | S | S | S | S | R | S | S | S | S | S | S | S | R | S |
| *S. uberis* | S | S | S | S | S | S | S | S | S | S | S | S | S | S | R | S |
| *S. uberis* | R | R | R | S | R | I | I | R | R | S | I | R | R | I | R | S |
| *S. uberis* | R | R | S | S | S | S | R | I | S | S | I | R | S | S | R | S |
| *S. uberis* | S | R | S | S | S | S | R | I | S | S | S | R | S | S | R | S |
| *S. uberis* | S | R | S | S | S | I | R | I | S | S | I | R | S | S | R | S |
| *S. uberis* | S | R | S | S | S | I | R | I | S | S | S | R | S | S | R | S |
| *S. uberis* | S | S | S | S | S | I | S | S | S | S | S | S | S | S | R | R |
| *S. uberis* | R | R | S | R | R | R | I | I | I | S | I | R | R | S | R | S |
| *S. agalactiae* | S | R | S | S | S | S | S | I | S | S | I | I | S | S | R | S |
| *S. agalactiae* | S | R | S | S | S | S | S | I | S | S | S | S | S | S | R | S |
| *S. agalactiae* | S | R | S | S | S | S | S | I | S | S | I | R | S | S | R | S |
| *S. agalactiae* | S | S | S | S | S | S | S | I | S | S | I | I | S | S | R | S |
| *S. agalactiae* | S | R | S | S | S | S | S | I | S | S | I | R | S | S | R | S |
| *S. agalactiae* | S | S | S | S | S | S | I | I | S | 8 | I | I | S | S | R | S |
| *S. agalactiae* | S | R | S | S | R | S | S | I | S | S | I | R | S | S | R | S |
| *S. agalactiae* | S | R | S | S | S | S | I | I | I | S | I | R | S | I | R | R |
| *S. agalactiae* | S | R | S | S | S | S | S | I | S | S | I | R | S | S | R | S |
| *S. agalactiae* | S | S | S | S | S | S | S | I | S | S | S | R | S | S | R | S |
| *S. agalactiae* | S | S | S | S | S | S | S | R | S | S | S | S | S | S | R | S |
| *S. agalactiae* | S | R | S | S | S | R | S | I | S | S | I | R | S | S | R | S |
| *S. agalactiae* | S | S | S | S | S | S | R | I | S | S | I | I | S | S | R | S |
| *S. agalactiae* | S | R | S | S | S | I | S | I | S | S | S | I | S | S | R | S |
| *S. agalactiae* | S | S | S | S | S | S | S | I | S | S | S | I | S | S | R | S |
| *S. agalactiae* | R | R | S | S | S | S | I | I | S | S | I | I | S | S | R | S |
| *S. agalactiae* | S | R | S | S | S | S | S | I | S | S | S | S | S | S | R | S |

AUG= amoxicillin and clavulanic acid, AMP= ampicillin, CFQ= cefquinome, CXM= cefuroxime, CZ= cefazolin; EFT= ceftiofur, E= erythromycin, ENR= enrofloxacin, FFC= florfenicol, IMI= imipenem, MAR= marbofloxacin, OT= oxytetracycline, PG= penicillin G, RAX= rifaximin, SXT= trimethoprim/sulphamethoxazole, VA= vancomycin.

**S-Table 2. Antibiotics susceptibility testing (AST) of Enterococcus spp. isolated from bovine with mastitis**.

| **Field Strains** | Antibiotics | | | | | | | | | | |
| --- | --- | --- | --- | --- | --- | --- | --- | --- | --- | --- | --- |
|  | AUG | AMP | E | ENR | FFC | IMI | MAR | OT | PG | RAX | VA |
| *Enterococcus sp.* | S | R | S | I | S | S | S | I | S | S | S |
| *Enterococcus sp.* | S | S | S | S | S | S | S | R | S | S | S |
| *Enterococcus sp.* | S | S | S | S | S | S | S | I | S | S | S |
| *Enterococcus sp.* | S | R | S | I | S | S | S | R | S | S | S |
| *Enterococcus sp.* | S | R | S | I | S | S | S | I | S | S | S |
| *Enterococcus sp.* | S | R | S | I | S | S | I | I | S | S | S |
| *Enterococcus sp.* | S | R | S | I | S | S | S | S | S | S | S |
| *Enterococcus sp.* | S | S | S | S | S | S | S | S | S | S | S |
| *Enterococcus sp.* | S | S | S | S | S | S | S | S | S | S | S |
| *Enterococcus sp.* | S | S | S | S | S | S | S | S | S | S | S |
| *Enterococcus sp.* | S | R | R | I | S | S | I | R | S | I | S |
| *Enterococcus sp.* | S | R | S | I | S | S | S | R | S | S | S |
| *Enterococcus sp.* | S | R | S | I | S | S | S | I | S | S | S |

AUG= amoxicillin and clavulanic acid, AMP= ampicillin, E= erythromycin, ENR= enrofloxacin, FFC= florfenicol, IMI= imipenem, K= kanamycin, MAR= marbofloxacin, OT= oxytetracycline, PG= penicillin G, RAX= rifaximin, SXT= trimethoprim/sulphamethoxazole, VA= vancomycin.

**S-Table 3. Antibiotics susceptibility testing (AST) of Staphylococcus aureus isolated from bovine with mastitis.**

| **Field Strains** | Antibiotics | | | | | | | | | | | | | | | | | | | |
| --- | --- | --- | --- | --- | --- | --- | --- | --- | --- | --- | --- | --- | --- | --- | --- | --- | --- | --- | --- | --- |
|  | AUG | AMP | CXM | CFQ | CZ | EFT | E | ENR | FA | FFC | FOX | G | IMI | K | MY | MAR | OT | PG | RAX | SXT |
| *S.aureus* | R | R | R | S | S | S | S | R | S | S | S | R | S | I | S | R | S | R | S | I |
| *S.aureus* | S | R | S | S | R | S | S | I | R | S | S | I | S | R | R | S | S | R | S | S |
| *S.aureus* | S | S | R | S | S | R | I | I | S | S | R | R | S | R | R | S | R | S | R | R |
| *S.aureus* | S | S | S | S | S | S | S | S | S | S | S | R | S | S | S | S | S | S | S | S |
| *S.aureus* | S | S | S | S | S | S | S | S | S | S | R | R | S | R | S | S | S | S | S | S |
| *S.aureus* | S | S | S | S | S | S | I | S | S | S | S | R | S | I | S | S | S | S | S | S |
| *S.aureus* | S | S | S | S | S | R | S | S | S | S | S | R | S | R | S | S | I | S | S | S |
| *S.aureus* | S | R | S | S | R | S | I | S | S | S | S | R | R | R | R | S | R | S | S | S |
| *S.aureus* | R | R | R | I | R | S | R | I | S | R | R | R | S | R | S | S | R | R | S | S |
| *S.aureus* | S | S | S | S | S | S | S | S | S | S | S | R | S | S | R | S | S | S | S | S |
| *S.aureus* | S | S | S | S | S | S | I | S | S | S | S | R | S | I | S | S | S | S | S | S |
| *S.aureus* | S | S | S | S | S | S | R | S | S | S | S | R | S | R | S | I | I | S | S | R |
| *S.aureus* | S | R | S | S | S | S | I | S | S | S | S | R | S | R | R | S | S | S | S | R |
| *S.aureus* | S | R | R | S | S | S | I | S | S | S | S | R | S | R | S | S | S | R | S | R |
| *S.aureus* | S | R | S | S | S | S | I | S | S | S | S | R | S | R | S | S | S | R | S | R |

AUG= amoxicillin and clavulanic acid, AMP= ampicillin, CXM= cefuroxime, CFQ= cefquinome, CZ= cefazolin, EFT= ceftiofur, E= erythromycin, ENR= enrofloxacin, FA= fusidic acid, FFC= florfenicol, G= gentamicin, IMI= imipenem, K= kanamycin, MY= lincomycin, MAR= marbofloxacin, OT= oxytetracycline, PG= penicillin G, RAX= rifaximin, SXT= trimethoprim/sulphamethoxazole.
